# An Evolutionary and Environmental Perspective of the Interaction of Nanomaterials with the Immune System

**DOI:** 10.3390/nano12060957

**Published:** 2022-03-14

**Authors:** Diana Boraschi

**Affiliations:** 1 Institute of Biochemistry and Cell Biology (IBBC), National Research Council (CNR), 80131 Napoli, Italy; diana.boraschi@ibbc.cnr.it; 2 Shenzhen Institute of Advanced Technology (SIAT), Chinese Academy of Sciences (CAS), Shenzhen 518055, China; 3 Stazione Zoologica Anton Dohrn, 80121 Napoli, Italy

Assessing the modes of interaction between engineered nanomaterials and the immune system is a topic of particular interest for research in several fields, from a toxicological and safety perspective to potential nano-based immunomodulatory strategies for medical use. This Special Issue gathers results and new information—mostly collected within the EU Horizon 2020 project PANDORA (probing the safety of nano-objects by defining the immune responses of environmental organisms), which specifically focuses on nano-immune interaction across living organisms, from plants to human beings. The underlying concept is that several of the immune defensive mechanisms used for tackling exogenous agents (including nanomaterials) are conserved across evolution with little modification. Thus, we looked for common mechanisms of recognition and reaction, based on the high evolutionary conservation of innate immunity, the ancient and highly efficient defensive system shared by all living organisms [1]. We wanted to find answers to the following questions:

1. Do nanomaterials pose threats to the organisms’ integrity or do the immune defensive mechanisms successfully deal with them?
2. Can we exploit our understanding of nano-immune interactions to devise nano-based tools to improve immune responses in vaccination?
3. Can we identify immune rections that are common across living organisms, and therefore we can use for a general nanosafety assessment of environmental and human health?

First question: **are nanomaterials seen by the immune system as a threat?** Yes, in some cases, in that the innate immune system, in particular phagocytes, can “see” the nanomaterials and begin action to eliminate the nanomaterials and maintain the organism’s functionality. Most interestingly, we should also consider the inverse interaction, i.e., how nanomaterials “see” the immune system and are modified by their interaction with it [2]. Notably, upon interaction, we can even observe beneficial biological effects, as in the case of decreased stress responses and growth promotion in the model plant *Arabidopsis thaliana* [3]. In many other cases, it is possible to observe an immune reaction, with morphological and functional changes in innate immune cells; however, these changes are not long-lasting and do not hamper the organism’s integrity. This means that a successful immune reaction has taken place, which has recognised the nanomaterials as a possible threat and has acted to eliminate them and re-establish normal tissue/organism functions [4,5,6,7]. A very important point that should be considered is that exposure to nanomaterials may affect immunity indirectly by interacting with immune-modulating entities. In particular, when nanomaterials are ingested, the interaction of nanomaterials with the resident microbiota must be considered, as microbiota are well known to shape intestinal and systemic immunity [8].

Second question: **can we use our knowledge of nano-immune interactions for designing “smart” nano-based vaccines**? The use of nanoparticles is a very promising approach to vaccination because the particles may double their scope by acting as a carrier for the vaccine antigens, being able to shuttle them preferentially to antigen-presenting cells while being active as an adjuvant, i.e., they are able to induce an innate/inflammatory response that is necessary for the optimal induction of a specific long-lasting immunity [9]. Two aspects have been considered here: the induction of a specific anti-infective protective immunity in poultry [10] and the possibility of modulating innate memory in human innate cells towards a more protective secondary response, thereby generating not only adaptive memory (resulting in enhanced secondary specific response) but also innate memory (i.e., a long-lasting amplification of the specific response) [11]. In both cases, nanoparticles derived from bacterial cells were used, and were able to act both as antigen carriers and adjuvant particles.

Third question: **can we design common assays that enable us to assess the cross-species effects of nanomaterials on immunity** (i.e., valid for both environmental species and human beings)? To this end, we have compared the most representative methods for evaluating immune reactivity across species in order to identify common conserved innate immune responses activated by interaction with nanomaterials. Excluding plants, whose extreme specialisation also impacts the type of immune defensive tools and responses, we have compiled a list of common assays, both in vivo and in vitro, that can be used to evaluate a response to nanomaterials (in terms, for instance, of safety) across animal species [12,13,14]. Notably, this implies that we can use some invertebrate models in vivo for predicting the effects of nanomaterials on human innate immunity [14].

To conclude, by examining immune response to nanomaterials across living organisms we have observed that immunity is, in general, able to cope with the nano-challenge and prevent detrimental effects to the organism. Both environmental species (marine and terrestrial invertebrates) and human beings display an array of common defensive mechanisms that are engaged in the interaction with nanomaterials, which allows us to identify model assays (in vivo and in vitro) which are predictive of nano-effects across species, making them useful for both environmental and human nano-safety testing. By evaluating nano-effects on the immune system, we can design nano-based vaccination strategies that exploit the immunomodulatory capacity of nanomaterials to achieve optimal long-term protective immunity.
